# Facilitators and barriers to the deprescribing of benzodiazepines and Z-drug hypnotics in patients under 65 on adult mental health wards

**DOI:** 10.1038/s41598-025-25261-4

**Published:** 2025-11-21

**Authors:** Sonia Filmer, Ian Maidment

**Affiliations:** 1 Tees, Esk and Wear Valley NHS Foundation trust, Lanchester Road, Durham, DH1 5RD UK; 2 Aston Pharmacy School, College of Health and Life Sciences, Aston University, Birmingham, B4 7ET UK

**Keywords:** Health care, Medical research

## Abstract

There is a place for the use of benzodiazepines/z-hypnotics on adult mental health wards, but they are often continued beyond a length of time where they are beneficial. This can result in dependence and withdrawal effects if stopped. Timely deprescribing of these medicines is encouraged, but there is limited evidence available as to what can be a facilitator or barrier to deprescribing benzodiazepines/z-hypnotics on these wards. Semi-structured interviews, with twenty-nine NHS healthcare professionals from eleven different NHS organisations, involved in the use of benzodiazepines/z-hypnotics on adult mental health wards, were conducted and recorded on Microsoft Teams and transcribed. Themes were generated via thematic analysis on NVIVO software, informed by a grounded, inductive approach, to identify similarities and differences in participants perceptions. The four main themes identified from participants’ experiences were: Culture (shared values, beliefs and practices). Patient factors. Practical measures to facilitate deprescribing of benzodiazepines/z-hypnotics on adult mental health wards. Primary/secondary care interface. Deprescribing culture, rather than being led nationally, is more influenced by local factors. Some patient behaviour patterns can be a barrier to deprescribing but many of these can be overcome by promoting patient-centred care, allowing patients to feel involved and enabled to make informed decisions around their care. Access to non-pharmacological methods to de-escalate behaviour, promote wellbeing and improve sleep on adult mental health wards can support deprescribing. Cohesive multi-disciplinary team working aids deprescribing, but staff pressures can hinder this by reducing access to appropriately trained staff. Effective discharge planning is important but not always achieved. Good communication post discharge can facilitate continued deprescribing. Overcoming the barriers and developing the facilitators identified could improve benzodiazepine/z-hypnotic deprescribing on adult mental health wards. Changes to national NHS culture and priorities are required to influence local culture. Otherwise, deprescribing practices will remain greatly influenced by local factors on individual wards. Good practice exists but further research and funding is needed to disseminate this throughout the NHS.

**Supplementary Information:**

The online version contains supplementary material available at 10.1038/s41598-025-25261-4.

## Introduction

Adult mental health (AMH) wards in the United Kingdom (UK) usually provide inpatient care, on single-sex wards, with intensive medical and nursing support for patients in periods of acute psychiatric illness^1^. Patients may be informal or subject to the Mental Health Act^2^. In 2023, the average length of stay on a UK AMH ward was 39 days^3^. Benzodiazepines (benzos) have been used for decades in the UK as anxiolytics and hypnotics on AMH wards^4^. Twelve benzodiazepines are licenced for use in the UK^5^. They slow down the central nervous system by acting as agonists at benzodiazepine receptors, enhancing the inhibitory effects of gamma-aminobutyric acid (GABA)^6^, resulting in calming and sedation. More recently, zopiclone and zolpidem (z-hypnotics), which also increase GABA transmission at these receptors, have emerged as alternative hypnotics^7^.

Despite a decline in prescribing levels, in 2021–22, 1.8% of England’s population without learning disabilities were prescribed benzodiazepines, rising to 7.1% among those with learning disabilities^8^. During 2017–18, 2.3% of adults in England were prescribed Z-drugs^9^. Benzodiazepines/z-hypnotics have established roles in treating mania, alcohol withdrawal and short-term anxiety relief and insomnia on AMH wards^10,11^. Nonetheless, they are often prescribed beyond their therapeutic window or inappropriately from the outset^9^.

National Institute for Heath and Care Excellence (NICE)^12^ guidelines recommend that benzodiazepines/z-hypnotics should not be prescribed for longer than 2–4 weeks to avert physical dependence and tolerance^5^. Between 15% and 44% of chronic benzodiazepine users may face moderate to severe withdrawal symptoms when discontinuing^13,14^, such as sleep disturbance, irritability, increased anxiety, panic-attacks, tremor, sweating and nausea^12^. Z-hypnotics cause dependence like benzodiazepines^9,15^ and withdrawal symptoms including insomnia, headaches, confusion, anxiety and restlessness^16^.

NICE concluded z-hypnotics offer no distinct advantage over benzodiazepines for treating insomnia^11^, despite a perception of increased safety regarding tolerance^17^. Prescribers’ often overestimate their benefits, neglecting inherent risks^18^. Continued treatment beyond four weeks necessitates a careful withdrawal plan^4,19^, which may distress patients and destabilise their recovery, necessitating additional primary care input. Reviewing benzodiazepines/z-hypnotics regularly^20^ can mitigate withdrawal symptoms. National Health Service (NHS) England’s guidance^21^ recommends medication reviews with the patient, should happen every 2–3 days throughout admission.

On AMH wards, benzodiazepines/z-hypnotics may be prescribed regularly or as-needed (PRN), with administration contingent upon nursing assessment of patients’ mental states. However, PRN administration practices reveal a lack of clarity and coherence^22,23^, with decisions often based on patient distress, safety concerns and requests. In some conditions, such as catatonia^24^, benzodiazepines/z-hypnotics are prescribed regularly. Compliance with NICE recommendations^12^ necessitates regular reviews and ideally stopping these medications before discharge, if not a withdrawal plan post discharge should be established.

Public Health England has recognised the urgent need for further research into benzodiazepines/z-hypnotics dependence^9^. In 2018, approximately 33% of patients discharged from AMH wards were on benzodiazepines/z-hypnotics^25^; with 20% continuing use 12 months post-discharge^8^. Deprescribing is “the process of tapering or stopping drugs, aiming to minimise polypharmacy and improve patient outcomes”^26^ Evidence for deprescribing is emerging from randomised controlled trials (RCT) and observational studies^27^. Challenges in the generalisation and transportability^28^ of RCTs makes it difficult to apply this evidence in the personalised care of NHS mental health settings^29^and evidence is limited in AMH inpatient settings. Facilitating deprescribing of benzodiazepines/z-hypnotics on AMH wards may reduce long term use of this medication post discharge.

Barriers to deprescribing stem from both clinician and patient perspectives, including prescriber confidence, communication issues and patient awareness^27,30^. Among primary-care prescribers, fears about consequences and workload concerns hinder action in patients under 65^27,31^. Procedural difficulties and perceived resistance are barriers with patients over 65^32–34^. There is more research into, and awareness of the benefits of, deprescribing of benzodiazepines/z-hypnotics in people over 65^35,36^than in the younger age group. As under 65s vary in recommended dosing levels and side-effect profile of benzodiazepines/z-hypnotics^5^, so it may not be appropriate to extrapolate findings from older age groups to AMH wards.

Prescribers underestimate deprescribing enablers, including patients’ concerns and experiences of adverse effects, dislike of multiple medicines^27^, staff and patient education^37–39^, a multi-professional approach^39^, acceptability of non-pharmacological alternatives^40^ and patient-centred care and shared decision making^40^. Person-centred care involves treating patients as individuals and as equal partners in their healing, meaning healthcare is coordinated, personalised and enabling^41^. A clinical audit in the Republic of Ireland showed that facilitators of benzodiazepines/z-hypnotic deprescribing on AMH wards include audit and feedback, increased clinical pharmacist input and placing limitations on prescribing^42^, but this audit is over 9 years old, is only from one hospital in a different healthcare setting and the research was not qualitative. New technologies may improve access to non-pharmacological treatments^11^.

In summary, the need for research into reducing benzodiazepines/z-hypnotics dependence^6^ is paramount, with limited evidence on deprescribing on AMH wards. This project aims to identify barriers and facilitators within deprescribing practices on AMH wards, setting the stage for future research and policy change that promotes effective management of these medications.

### Aims

To identify barriers and facilitators, perceived by healthcare professionals, to deprescribing of benzodiazepines/z-hypnotics on AMH wards.

### Objectives

To interview healthcare professionals to investigate current practices in reviewing and deprescribing and identify barriers and facilitators associated with deprescribing benzodiazepines/z-hypnotics on AMH wards.

### Method

This exploratory qualitative study involved remote interviews, adhering to the Consolidated Criteria for Reporting Qualitative studies (COREQ) guidelines^43^. A copy of the completed COREQ guidelines are available in the supplementary information file section.

### Ethics

Ethical approval was secured from the Health Research Authority (IRAS number 322529) and Aston University’s Governance Committee. Additionally, capacity and capability approvals were obtained from Tees, Esk and Wear Valley NHS Foundation Trust. Clinical trial number: not applicable. All methods were performed in accordance with the relevant guidelines and regulations.

### Inclusion criteria

Healthcare professionals currently or recently (within the last 6 months) working on NHS AMH wards in the UK, with experience using or reviewing benzodiazepines/z-hypnotics in patients aged 18–65.

### Exclusion criteria

Healthcare professionals not working on NHS AMH wards in the UK within the last 6 month or lacking experience using or reviewing benzodiazepines/z-hypnotics in patients aged 18–65.

### Participants and recruitment

A combination of convenience, snowball and purposive sampling techniques was employed to recruit participants through NHS contacts and professional networks. Convenience sampling involved the researchers asking existing contacts from their workplaces to take part in interviews or to promote the study among their contacts using an advertising flyer. A copy of the advertising flyer is available in the supplementary file section. Purposeful sampling was performed by sharing social media posts through the College of Mental Health Pharmacists network. Participants were encouraged to disseminate information within their networks, which resulted in a snowball effect.

### Interview process and consent

Interviews took place from November 2023 to March 2024 and were all carried out by the same researcher. Pilot interviews with two healthcare professionals were successfully conducted, with no modifications needed, and included in the final dataset. A participant information sheet was sent out, along with consent and demographic data forms to complete prior to interviews. Copies of the participant information sheet, consent form and demographic data form are available in the supplementary file section. The interviewees had the opportunity to ask questions and confirm their consent before beginning the interview.

Semi-structured interviews were conducted online, recorded and transcribed using Microsoft Teams. This transcription was anonymised and accuracy checked by the same researcher who had carried out the interviews. An interview guide facilitated flexible discussions, allowing for spontaneous questions and in-depth participant reflections. A copy of the interview guide is available in the supplementary file section. This heuristic approach^44^ enabled richer data collection while supporting systematic comparison across interviews.

### Data analysis

Transcribed interviews were coded and analysed using NVivo software, employing thematic analysis^45^ with a grounded^46^, inductive approach. This allowed for open coding and opportunities to draw out meanings expressed by interviewees. Continuous data analysis enabled flexibility in interviews to explore emerging themes^44,47^ whilst identifying data saturation^45,48^, which was achieved after 29 interviews, signalling an end to participant recruitment.

### Reflexivity and validation

The researcher, a mental health pharmacist with over 25 years of clinical experience, remains mindful of her dual role as researcher and clinician. Reflexivity informed the analysis, ensuring participants’ views were conveyed accurately, accounting for potential biases from professional relationships^49^ and experiences. Dissenting opinions were analysed and opposing views reported.

## Results

Participant demographics are shown in the Table 1 below.

**Table 1:** Participant demographics.

Participant ID	Role	Prescriber?	Anonymised number given to reflect which NHS trust the participant worked in over the last 6 months	Area of UK worked in
Doctor1	Senior registrar	Yes	1&3	Northeast England
Doctor2	Consultant psychiatrist	Yes	1	Northeast England
Doctor3	Consultant psychiatrist	Yes	3	Northeast England
Doctor4	Senior registrar	Yes	5	West Midlands England
Doctor5	Consultant psychiatrist	Yes	1	Northeast England
Doctor6	Consultant psychiatrist	Yes	1	Northeast England
Doctor7	Senior registrar	Yes	1	Northeast England
Nurse1	Mental health nurse	No	1	Northeast England
Nurse2	Mental health nurse	No	2	Wales
Nurse3	Mental health nurse	No	1	Northeast England
Nurse4	Consultant nurse and approved clinician	Yes	1	Northeast England
Nurse5	Consultant nurse and approved clinician	Yes	1	Northeast England
Nurse6	Consultant nurse and approved clinician	Yes	11	Yorkshire
Pharmacist1	Clinical pharmacist	Yes	1	Northeast England
Pharmacist2	Clinical pharmacist	Yes	2	Wales
Pharmacist3	Clinical pharmacist	No	3	Northeast England
Pharmacist4	Clinical pharmacist	Yes	4	East Midlands
Pharmacist5	Clinical pharmacist	No	3	Northeast England
Pharmacist6	Clinical pharmacist	No	3	Northeast England
Pharmacist7	Clinical pharmacist	Yes	6	Northwest England
Pharmacist8	Clinical pharmacist	No	4	East Midlands England
Pharmacist9	Clinical pharmacist	Yes	4	East Midlands England
Pharmacist10	Clinical pharmacist	Yes	7	Northwest England
Pharmacist11	Clinical pharmacist	No	7	Northwest England
Pharmacist12	Consultant pharmacist	Yes	8	Southwest England
Pharmacist13	Clinical pharmacist	No	4	East Midlands England
Pharmacist14	Clinical pharmacist	Yes	9	Wales
Pharmacist15	Clinical pharmacist	No	10	Northwest England
Pharmacist16	Clinical pharmacist	Yes	3	Northeast England

29 healthcare professionals meeting the inclusion criteria were interviewed from 11 different NHS organisations: average of 4 years in their current roles and 12.9 years’ experience in mental health. 79% identified White; 14% Asian: 3% mixed or from multiple ethnic groups; 3% as other ethnicities. 55% were female. Interviews lasted 20–50 min. Four main themes emerged: culture, patient factors, practical measures to facilitate deprescribing of benzodiazepines/z-hypnotics on AMH wards, and primary/secondary care interface.

## Culture

### National and organisational culture and agenda

Clinicians felt isolated due to a lack of standardisation of guidelines and clinical practice nationally leading to inconsistencies in prescribing cultures across different sites, leading to large variations in the frequency benzodiazepines/z-hypnotics were initiated, administered and deprescribed. *“I’ve seen various strategies and good individual practices*,* but it’s not systematic or standardised enough. The right culture isn’t established yet.’(Doctor1)*.

Although participants described that the Ashton Manual^50^, commonly used in the UK for understanding and managing benzodiazepine withdrawal, and NICE document^12^, evidence-based recommendations aiming to improve health in the UK, were useful to support them to deprescribe benzodiazepines and hypnotics, awareness of the content of the NICE guidelines was limited. Participants requested more accessible resources, such as national guidelines aimed for use on AMH wards, compiling all necessary documentation necessary to create patient-specific deprescribing plans. Area-Prescribing Committee benzodiazepine/z-hypnotic withdrawal guidelines helped continuity of care between AMH wards and primary-care. Many were unaware if local guidelines existed in their organisation.

Some interviewees described how Care Quality Commission (CQC) monitoring and national prescribing targets improved benzodiazepine/z-hypnotic deprescribing culture in some organisations, reporting how GPs and community mental health teams (CMHT) sometimes resisted taking on benzodiazepine/z-hypnotic prescribing when included in prescribing targets. *“Some community teams emphasise they wouldn’t support patients on benzodiazepines*,* facilitating easier implementation.”(Doctor5).* Subsequently, benzodiazepines/z-hypnotics were halted post-discharge unless the ward provided compelling rationale for their continuation.

Leadership promoting integration of deprescribing into organisational culture, including encouraging incident reporting, audit and quality-improvement, aided by electronic prescribing systems (EPMA), was felt by some participants to incentivised change. *“Leaders at a strategic level must apply sufficient pressure to address existing problems around benzodiazepine prescribing*,*”(Pharmacist1) “I train primary-care pharmacists to incident report if hospitals persistently discharge people on benzos.”(Pharmacist10*.) *“Good audit illustrates the absence of patient activity during evenings*,* correlating with self-harm and subsequent lorazepam use.”(Doctor4)*.

Some participants felt educating society nationally on harms of long-term benzodiazepines/z-hypnotics use and altering cultural expectations may facilitate deprescribing. Others thought the smokefree culture in hospitals may contribute to continued benzodiazepine/z-hypnotic use, sometimes mitigated by proactivity around nicotine-replacement therapy. *“Patients are already anxious and not sleeping. If anxious at home*,* they smoke…maybe thinking about smoking policy?”(Doctor1)* Two interviewees supported increasing national legislation on prescribing and administration of benzodiazepines/z-hypnotics may support deprescribing. *“They would be less utilised by staff members if treated like controlled-drugs…*”(Pharmacist9).

### Ward culture

Attitudes and actions of consultants, ward managers, nursing staff, and pharmacists was mentioned by most participants as being important in shaping local prescribing practices. *“Different wards have different managers and different experiences.”(Pharmacist11)* Consultants prioritising reviews helped deprescribing. Junior doctors’ training and confidence disproportionately affected the culture; over-prescribing on admission complicated later deprescribing. *“Our current medics don’t have the same awareness around benzos and hypnotics. Once prescribed it’s difficult to say no.”(Pharmacist6)*. Better defined indications for appropriate benzodiazepines/z-hypnotics and review/stop dates aided deprescribing. On-call prescribers often struggled using EPMA systems, leading to unnecessary continuation of PRN medication. *“Nurses frequently wish to retain PRNs because there’s a problem with on-call medics using EPMA.”(Pharmacist12)*.

Collaboration and consistency among staff enhanced deprescribing culture. *“Medication seeking patients ask staff members they know will give it. Everyone must be consistent.”(Nurse1)*. Staff training and experience impacted this; inexperienced medics were reluctant to reduce benzodiazepines/z-hypnotics, inexperienced pharmacists did not challenge prescribing; inexperience nurses struggled to encourage non-pharmacological methods while confident nurses supported deprescribing. *“Nurses question what patients want the meds for*,* do they actually need it?”(Pharmacist11) Conversely*, doctors reported nurses discouraged deprescribing. “Nurses *are clear to Junior Doctors they want prn’s prescribed just in case.”(Doctor1)* Many staff relied on informal shadowing rather than formal training. *“We’ve nothing formal in place. New members of staff shadow and follow us around.”(Pharmacist2)*.

## Patient factors

### Patient behaviour

Increasingly violent, self-harming patient behaviours caused concerns amongst interviewees about managing escalating behaviours without benzodiazepines/z-hypnotics hindering deprescribing. *“The last three years has seen elevated violence*,* aggression and self-harm.”(Nurse5)*.

Many of the AMH healthcare professionals interviewed reported patients’ dependent on benzodiazepines/z-hypnotics showed reluctance to reduce them. *“Patients like taking them as many use street Valium; prefer a prescription rather than buying it.”(Nurse2).* Nurses struggled policing benzodiazepines/z-hypnotics in patient who abuse illicit drugs. *“It would be easier stopped in patients who abuse them…then nurses don’t have to decide whether to give benzos or hypnotics*,* and patients cannot pressurise to have.”(Nurse1).*

Interviewees observed that patients admitted with sleep-deprivation were sometimes reluctant to discontinue hypnotics when their sleep improved, fearing relapse. Nurses particularly commented on the effect that disrupted daily routines and behaviour patterns had on hypnotic deprescribing *“…patients often get a massive take away at 9pm. They’re not going to sleep easily after that. We aren’t allowed to stop that behaviour.”(Nurse1)*.

Some participants felt patients could be manipulative, which impacted deprescribing. *“When the MDT deprescribe*,* patients discuss amongst themselves how to get medication. They know what to say to get it.”(Nurse1)*.

### Past medical history and indication

Deprescribing benzodiazepines/z-hypnotics was reported by some interviewees to be easier in patients with clearly defined mental health diagnoses, facilitated by structured treatment pathways. Those prescribed higher, regular doses for conditions such as catatonia often experienced more managed deprescribing processes than those on PRN.

The absence of alternative treatments appeared to hinder deprescribing, although promethazine was sometimes used. *“…promethazine first line*,* having a tier option.”(Doctor 5)* Deprescribing proved challenging for a number of interviewees in patients with emotionally unstable personality disorder (EUPD), especially with pre-existing polypharmacy, due to the perceived, and actual, risk of severe self-harm. *“…tricky group of female EUPD patients…been there for months and collected multiple medications*,* including benzos. The self-harm with EUPD…has meant an overreliance on pharmacology.”(Pharmacist 12)*. A history of alcoholism or severe agitation was also a barrier.

Long admissions provided opportunity to support the patient to withdraw benzodiazepines/z-hypnotics. Conversely, if benzodiazepines/z-hypnotics were started during a long admission and dependence developed, then participants mentioned deprescribing was complicated by the need to slowly withdraw and fear over jeopardising recovery.

### Person-centred care

Some AMH healthcare professionals interviewed felt shared-decision making was necessary for deprescribing benzodiazepines/z-hypnotics, *“I’ve seen prescribers stop it*,* but not explain it to the patient*,* who asks the nurse for it and it’s re-prescribed.”(Pharmacist 11)*. Weekly multi-disciplinary team (MDT) ward rounds, including patient participation, enabled effective, actionable care-plans to be documented including de-escalation techniques.

Strong relationships between nursing staff and patients was reported by participants to reduce reliance on benzodiazepines/z-hypnotics. Person-centred care enabled prescribers build patient trust and understanding, facilitating deprescribing. Sometimes fear of damaging prescriber: patient relationships inhibited review. *“You fear telling someone you’re going to stop their benzos in case you fracture the relationship.”(Doctor 3)*.

## Practical measures to facilitate deprescribing benzodiazepines/z-hypnotics on AMH wards

### MDT working

Good access to MDT professionals facilitated safe reduction of benzodiazepines/hypnotics. Higher nursing staff levels reduced reliance on benzodiazepines/z-hypnotics. “*…higher nurse: patient ratios so didn’t need to pharmacologically manage people in the same way.”(Pharmacist 13)*.

Pharmacists appeared to more pick up on the role of the extended MDT, especially Psychologists and Occupational Therapists, in supporting deprescribing. *“Psychologists are very useful in someone using lots of benzos. They speak to them.”(Pharmacist 11)*. All professions appeared to value the role of clinical pharmacists and non-medical prescribers (NMP) to facilitate deprescribing. “*….drawn upon pharmacists’ expertise to have conversations with patients about stopping benzodiazepines and hypnotics.*”(Nurse6). *“It’s really positive having more non-medical prescribers in advanced roles on adult inpatient.”(Doctor1)* Healthcare assistants, peer-support workers, experts-by-experience, activity coordinators and gym instructors lessened benzodiazepine/hypnotic reliance through non-pharmacological interventions such as exercise, especially in sectioned patients without unaccompanied leave.

All the professions interviewed agreed that staffing pressures constrained staff effectiveness. *“If patients were busier with better ward activities and activity coordinators not used like taxi drivers*,* prn use would reduce.”(Doctor 1)*. It was perceived staffing levels were worsening. *“We’re running into problems post COVID with lack of staff. It’s an ongoing barrier.”(Pharmacist 10)* This was compounded if multiple complex patients were on the ward. *“When there’s high acuity on the wards and limited staffing*,* unfortunately PRN medication becomes the easier option.”(Doctor 2)* Inconsistent staffing affected building strong patient relationships. Pharmacists seemed more concerned than nurses or doctors over benzodiazepine/hypnotic reviews being frequently cancelled due to staffing, or key staff being unavailable to take part. Staff availability for emergency response caused concern. *“There isn’t the staff around on other wards to respond*,* so we reach for benzos*,* which is not necessarily inappropriate*,* if it keeps staff and patients safe.”(Pharmacist 12)*.

Prescribing review meetings could help. *“Other trusts should introduce prescribing reviews. It’s the main way we reduce benzos because we’ve got specific allocated time.”(Pharmacist 6)* EPMA systems and utilising video conferencing, allowing remote working, enabled MDT review. Templates organising and recording MDT discussions added extra evidence to deprescribing decisions.

### Non-pharmacological support

Multiple interviewees mentioned talking to patients facilitated deprescribing by promoting verbal de-escalation and reducing PRN use, however, different resources appeared to be used including talking therapies and the talk-first initiative^51^,*“talk-first initiative encourages use of verbal de-escalation instead of offering PRN first….the patient can be challenged and have boundaries set….”(Pharmacist 5)*.

There was a great deal of variation in which non-pharmacological methods Interviewees felt was effective to reduce patients’ reliance on benzodiazepines. Those mentioned included breathing exercises, distress tolerance, grounding techniques, mindfulness, distraction techniques, relaxation methods, muscle stimulation therapy and cognitive behavioural therapy. Anti-anxiety boxes containing craft items and sensory equipment, and calm cards identifying patient-led plans to reduce agitation prior to PRN, facilitated deprescribing. *“…fidget spinners*,* watching cat videos*,* going for a walk…an individualised plan that helps staff know what they can do to support that patient.” (Pharmacist 15).* Variation in which methods were mentioned appeared to be more related to which trust the interviewee worked for, rather than which profession they were from. Many seemed to agree that non-pharmacological methods should be individualised and agreed in advance with the patient.

Sleep-hygiene leaflets/sleep-packs were not always given to patients or AMH ward staff reported patients dismissed them as ineffective. *“We will counsel people on sleep-hygiene. It generally doesn’t go down well*,* people feel they’re being patronised.” (Pharmacist 15)*. MDT out-of-hours support facilitated effective sleep-hygiene. Participants reported some patients stayed up late drinking caffeinated and energy drinks, then requested hypnotics; many wards did not supply caffeinated drinks. Inpatient access to addiction services and support groups around benzodiazepines/z-hypnotics was lacking. *“There’s no facility within substance misuse services*,* no support groups around prescribed benzodiazepines and z-drugs.” (Pharmacist 8)*.

### Ward environment

Facilities to support patients to use less benzodiazepines/z-hypnotics varied. *“…ward’s got gym equipment as a way to de-stress rather than using the benzos.” (Pharmacist 11)* A quiet room where people go to calm down was useful. *“We’ve a snoozelum. It’s a relaxation room with nice lights and music on.” (Pharmacist 14)* Many wards lacked sufficient private areas. *“Our ward doesn’t have spaces for conversations about medications.” (Nurse 6)*.

Interviewees observed wards could be unsettling environments for patients, due to noise and behaviour of other patients. *“Reviewing benzos and hypnotics usually gets missed because of how chaotic the ward is. Patients get left on medication when the clinical need isn’t there.” (Nurse 3)*.

AMH healthcare professionals felt a lack of “home-comforts”, uncomfortable beds and overnight staff observations disturbing sleep were barriers. Participants from one organisation felt their Sleep Well^52^ scheme reduced hypnotic use by reducing disturbances overnight.

## Primary/secondary care interface

### Leave and discharge planning

Planning benzodiazepine/z-hypnotic tapering before or during patient leave was crucial for reinforcing deprescribing. *“If the patient manages without benzos or hypnotics on leave*,* it breeds confidence they don’t need PRN’s.” (Pharmacist 6)* Lack of structured leave plans often resulted in patients continuing benzodiazepines/z-hypnotics unchanged during leave. Patient involvement seemed important. *“Junior medics tend to do leave scripts; discussions aren’t always had between the wider team and with the patient. They go on leave with the prn’s they took on the ward.” (Doctor 7)*.

Rushing deprescribing during discharge risked destabilising the patient or causing withdrawal symptoms. Implementing deprescribing treatment-plans prior to discharge helped, but patients were sometimes discharged in the middle of this plan, often because of bed-pressures or self-discharge. Benzodiazepine/z-hypnotic deprescribing may not be prioritised during discharge planning. *“I don’t think it’s considered. Priorities are suicide prevention planning or getting the patient food. Benzos are falling off the bottom of the list.” (Pharmacist 10)*.

### Communication on discharge

AMH healthcare professionals interviewed indicated effective discharge communication ensured ongoing deprescribing post-discharge. Discharge letters including a benzodiazepine/hypnotic reduction plan facilitated further management in the community. Oten discharge letters were inaccurate in recording benzodiazepine/z-hypnotic use, but pharmacy checks may improve this. *“Benzos and hypnotics are started as an inpatient and intended for short-term use*,* but it’s not communicated well to GPs (General Practitioners)*,* so inappropriately added on GP repeats.” (Pharmacist 13)* Discharge counselling and discussing benzodiazepine/z-hypnotic prescribing in discharge meetings facilitated deprescribing.

Usually only small quantities of benzodiazepines/z-hypnotics were provided on discharge. This should be explained to the patient and documented. Some organisations provided larger amounts, complicating deprescribing. Without a written plan, interviewees felt GPs sometimes lacked confidence to reduce medications and CMHTs struggled with capacity to support continued deprescribing. *“GPs are reluctant to review or stop these meds as they were started by a specialist.” (Pharmacist 13).* Participants felt one national healthcare computer system would facilitate communication between sectors.

## Discussion

### Summary

Significant variation persists in the management of benzodiazepine/z-hypnotics on AMH wards. Local ward culture, rather than national guidelines, chiefly drives the approach to deprescribing, compounded by some patient behaviour patterns which can hinder this process. Adopting patient-centred care can alleviate many of these challenges. Access to non-pharmacological interventions aimed at de-escalating agitation, enhancing wellbeing, and improving sleep are crucial in supporting benzodiazepine/z-hypnotic deprescribing. Collaborative MDT efforts can bolster this initiative, although staff pressures are often a barrier. Effective discharge planning that prioritises safe deprescribing is vital but often overlooked due to bed pressures. If benzodiazepines/z-hypnotics are continued post-discharge; good communication with CMHTs/primary-care facilitates continued deprescribing.

### Detailed discussion

This research found the dominance of local culture influencing deprescribing of benzodiazepines/z-hypnotics on AMH wards leads to discrepancies in treatment pathways. Allowing local culture to influence this deprescribing may lead to a clinician-centred medical culture, which has been highlighted as a barrier to deprescribing^40^. Deprescribing has been described as “swimming against the tide” owing to patient expectation, entrenched medical norms and organisational constraints^53^. A strong national agenda around deprescribing benzodiazepines/z-hypnotics on AMH wards is needed to help individual clinicians have the support they need to overcome these constraints.

Although many clinicians referred to the Ashton Manual^50^ for guidance, it’s focus is primarily on community settings, leaving a gap in support for inpatient scenarios. Brandt^54^ highlighted inconsistencies and/or insufficiency of detail among deprescribing documents for benzodiazepines/z-hypnotics. Due to the individual nature of benzodiazepines/z-hypnotic deprescribing, resources need to be flexible so the patient can guide adjustments^12^. Research on deprescribing benzodiazepines/z-hypnotics tends to focus on over 65s or on deprescribing in primary care^36,37^. A national resource combining inpatient guidelines for benzodiazepines/z-hypnotics prescribing, withdrawal regimes, patient-information leaflets and non-pharmacological support could support the process. National resources are available in Wales^55^ and Scotland^56^ to support appropriate benzodiazepine/z-hypnotic prescribing, but not specifically for inpatients.

Barriers to healthcare professionals accessing evidence-based guidelines include time constraints, lack of awareness, guideline complexity and disagreement^57^. Any new resources should be easy to use, with effective training and resource dissemination targeted to the AMH inpatient environment^55^. Initiatives such as Area Prescribing Committee guidelines can facilitate continuity of care, while the new NICE insomnia^11^ guidelines may inform future practices, depending on their promotion and adoption.

Multidisciplinary ‘deprescribing committees’ aid deprescribing in psychiatry^58^ but motivation and training of MDT members affects successful deprescribing^34^. Capability barriers exist, especially among out-of-hours medical staff. Organisations need to ensure that on-call medical staff have the knowledge and IT skills required to safely prescribe and deprescribe. Simple educational strategies can reduce hypnotic prescribing rates and enhance staff confidence in insomnia management on mental health wards^25^.

A dedicated team member, often a clinical pharmacist, overseeing the deprescribing process facilitated successful outcomes, mirroring findings elsewhere^59,60^. Hawkins^61^ recommends using pharmacists to facilitate communication between prescribers, communicate risks to patients, and implement tapering/discontinuation plans. However, pharmacist confidence and capacity may impede effective implementation^62^. NMPs may enhance benzodiazepines/z-hypnotic management on AMH wards in a similar way to their role in primary care, by leveraging their detailed medication knowledge^63^ and improving access to prescribers. The role of experts-by-experience in medicines optimisation has been documented^64^ but research is needed into their role in deprescribing.

Time pressures and pressures to discharge limit benzodiazepine deprescribing^34^. In 2024, CQC identified staffing shortages as one of the greatest challenges for the mental health sector, impacting quality and safety of care^65^. Reports from 39 UK mental health trusts, referencing staff shortages as a contributing factor to serious incidents, rose from 6957 in 2019 to 11,073 in 2022^66^. There was an increase in restrictive practices on AMH wards associated with high staff sickness during the COVID pandemic^67^. Patients detained under the Mental Health Act may be denied escorted section 17 leave due to staff shortages^68^. Time away from the ward and exercise can be used to manage sleep-hygiene^11^ and de-escalate aggression^51^. Poorly managed patient aggression negatively impacts healthcare workers’ psychological well-being and staffing levels^69^, causing a spiral where less staff are available to offer non-pharmacological management of aggression, resulting in less deprescribing of benzodiazepines/z-hypnotics. Staff fears over managing patient aggression without benzodiazepines/z-hypnotics is an example of fear contributing to deprescribing inertia, which is not easily allayed by limited evidence regarding safety and efficacy of deprescribing^70^.

Patient-centred approach enables deprescribing^32^ but often requires collaborative engagement among professionals^71^. Patients are more amenable to deprescribing conversations if they understand the rationale, are involved in developing the tapering plan, and offered behavioural advice^34,72^. Educating patients around deprescribing decisions prevents damaging patient/prescriber trust^73^. Staff following the safe wards model^51^ reported increased confidence to use de-escalation skills before offering benzodiazepines/z-hypnotics. Trauma-informed care has been shown to reduce incidents of self-harm, seclusion and restraint on AMH wards^67^. Benzodiazepine/z-hypnotic deprescribing would be facilitated if further research highlighted which de-escalation techniques were most effective on AMH wards, resulting in national standardisation. Using EPMA systems to give prompts can aid deprescribing^53^ however, existing systems may require adaption for diverse healthcare settings^74^.

Contrary to the negative views around sleep-hygiene advice in this research, advising on sleep-hygiene has been associated with improved mental health outcomes^75^. Research is needed^76^ into how to tailor this to the individual^77^ as NHS patient-information leaflets are sometimes inaccurate, inconsistent and confusing^78^. Sleep-hygiene, when part of a larger quality improvement bundle, has been associated with a sustained reduction in hypnotic prescribing in general hospitals^79^. Caffeine use by patients was highlighted as a barrier to deprescribing benzodiazepines/z-hypnotics on AMH wards by some interviewees and although the negative effects of caffeine on sleep are well documented^80^ and concern raised over the neurovegetative effects energy drinks^81^, further research is needed into their effect on insomnia and agitation on AMH wards. The Sleep Well programme^52^, mentioned by some interviewees as having a positive impact on deprescribing benzodiazepines/z-hypnotics on AMH wards, studied contributory factors to poor sleep on psychiatry wards; protecting sleep with reduced overnight checks personalised to the patient.

Establishing and documenting clear rationale for initiating benzodiazepines/z-hypnotics is important as they are not always indicated^82^. The BNF^5^ advises caution in initiating benzodiazepines/z-hypnotics in patients with a history of drug or alcohol abuse. If prescribed inappropriately, identifying the point at which deprescribing can be considered is made more difficult. Effective communication with patients can be impaired during admission, due to illness-related factors, acute intoxication and unwillingness to communicate^83^ so a conversation on the short-term use of benzodiazepines/z-hypnotics may need to be delayed.

Some participants anecdotally felt that gender may be a barrier to deprescribing benzodiazepines/z-hypnotics on AMH wards, particularly mentioning female patients with EUPD. Research has shown no difference between the genders in terms of aggression^84^, but more female patients are admitted with EUPD than male^85^. Peters^86^ found being male significantly decreased the likelihood of receiving benzodiazepines at discharge from psychiatric inpatient treatment. Concern has been raised over the lack of evidence in prescribing in EUPD^85^, resulting in a large burden of psychotropic polypharmacy^87^. Benzodiazepine prescribing in EUPD increased from 55.3% in 2014 to 58.6% in 2019^87^. At times of crisis, few patients with EUPD engage in psychotherapies^85^ and specialist individualised services are required^85^. Future research should identify the support required for people with EUPD, on AMH wards, to reduce benzodiazepine/hypnotic use and to investigate if behavioural differences exist between the genders, or if it reflects therapeutic nihilism from ward staff.

Although some participants mentioned using Promethazine as an alternative to support deprescribing of benzodiazepines/z-hypnotics, it’s use is associated with several adverse-effects^5^ and has little support in current NICE guidance^11^. NICE recently recommended daridorexant for chronic insomnia^11^. This novel hypnotic may facilitate benzodiazepine/z-hypnotic deprescribing by offering an alternative treatment^88^. Research on elderly wards concurred that long admissions facilitated deprescribing in patients admitted on benzodiazepines/z-hypnotics but acted as a barrier to deprescribing in patients started on these medications during admission^89^.

Benzodiazepine/z-hypnotic deprescribing not being prioritised or completed at discharge mirrored previous research^90^. More research into discharge priorities from AMH wards could help us understand the priority given to benzodiazepine/z-hypnotic deprescribing. If deprescribing plans were put in place during initiation of benzodiazepines/z-hypnotics and well documented, then these plans could be included in the discharge communication.

Some of the AMH healthcare professionals interviewed had observed that primary-care/CMHT influenced deprescribing practices on AMH wards by applying additional barriers to discharging patients on benzodiazepines/z-hypnotics. This would appear to follow the behavioural change wheel to implement evidence-based practice^91^. Mitchie^91^ found motivation is one of the components of behavioural change and can take the form of incentivisation or coercion. Incentivising primary-care to reduce their benzodiazepine/z-hypnotic prescribing may have resulted in some coercion of secondary-care to encourage deprescribing. However, discontinuation post discharge should be discussed with the patient, and planned for early on in treatment, to prevent distress or withdrawal effects post-discharge.

Concern raised by some participants over the inaccuracies in information on benzodiazepine/z-hypnotic deprescribing in discharge letters seems to be in line with research by Keers^92^ who found discharge prescriptions issued by mental health NHS hospitals have high levels of prescribing, clerical and communication errors. If accurate deprescribing information is not provided by the AMH ward on discharge, primary care clinicians may be hesitant to stop a medication started by a consultant psychiatrist as they do not feel responsible for the medication, because of professional hierarchy^21^. Direct communication between healthcare professionals when considering deprescribing is invaluable to resolve uncertainties, prevent conflicting instructions to patients, and ensure alignment of the therapeutic plan^93^. Creating a culture that values communication at discharge helps improve outcomes following hospitalisation^94^. Ensuring primary and secondary care have access to a universal patient note and prescribing system would aid deprescribing by passing information efficiently between sectors^95^.

Although many of the barriers, such as prescriber confidence and workload pressures^27,30,31^ and facilitators, such as staff and patient education, MDT approach and non-pharmacological alternatives^37–40^,identified in this study correlate with findings from previous research on deprescribing in other settings, some of the finding of this study appear to be unique to the setting of deprescribing benzodiazepines/z-hypnotics on AMH wards. Deprescribing culture being acutely influenced by local, ward factors, rather than a strong national agenda with robust guidelines aimed at AMH wards was an important barrier identified in this study. As was the importance of good discharge planning and communication on discharge to enable deprescribing of benzodiazepines/z-hypnotics prior to or post discharge.

### Strengths and limitations

The large numbers interviewed from multiple organisations is a strength of the study. Although participants were recruited throughout England and Wales, a large proportion were from the northeast of England, so regional differences may have skewed the results. Participants recruited via convenience sampling and snowball effect from contacts of the researcher may not be representative of the population. Interviewees putting themselves forward for interview may have already had an agenda they wished to pursue on this topic or an interest in the subject, resulting in volunteer bias^96^.

The researcher’s clinical role as a pharmacist on AMH wards may have affected the discussions with participants and interpretation of the results. To overcome interviewer bias^97^ the researcher aimed to be transparent and reflexive throughout. Pharmacists were overrepresented in the demographics, with nurses being the least represented group. The absence of patient interviews was also a limitation. Future research should include a wider range of healthcare professionals and patients.

A small number of interviewees worked directly with the researcher, which may influence the answers they gave. The researcher tried to avoid confirmation bias^98^ by considering all data obtained while analysing and re-evaluating and discussing this with the chief investigator. Member checking of transcripts was not carried out, which may have led to misinterpretation during some of the transcribing.

### Implications for policy makers

A strong national agenda is needed to drive a local culture of deprescribing benzodiazepines/z-hypnotics on AMH wards, including developing a national evidence-based resource which can support this. On-call medic cover on AMH wards should have the knowledge and IT skills to safely prescribe. EUPD treatment recommendations should be updated to provide more guidance on use of medication and access to specialist resources. Current staffing levels, and jobs descriptions of staff, on AMH wards should be reviewed to ensure benzodiazepines/z-hypnotic deprescribing can be supported safely. The ability of sectioned patients to access section 17 leave should be audited. Sharing of good practice between mental health trusts should be encouraged.

### Implications for future research

Qualitative research into patient views around deprescribing benzodiazepines/z-hypnotics and research into factors which resulted in patients being discharged on these medications, would allow for triangulation. Qualitative research into the role played by various MDT members in deprescribing may identify changes in job descriptions and further training needs. Further research to identify the most effective de-escalation and sleep-hygiene techniques, would enable evidence-based national standardisation. Research is needed into the effect of energy drink consumption on insomnia and agitation on AMH wards, to see they affect patient behaviour and act as a barrier to benzodiazepine/z-hypnotic deprescribing. Further research is required to observe if gender differences exist in prescribing/deprescribing patterns of benzodiazepines/z-hypnotics in patients diagnosed with EUPD. Research into discharge priorities from AMH wards may indicate if deprescribing is considered during transfer of care.

## Conclusion

Identifying facilitators and barriers to deprescribing benzodiazepines/z-hypnotics on AMH wards may be a first step on the pathway to alleviate the persistent issue of these medications being continued post-discharge, risking tolerance, dependence and withdrawal symptoms. While patient-specific barriers related to behaviour and motivation exist, they can be mitigated with non-pharmacological treatments and support from well trained staff. Maintaining adequate staffing across the full MDT is crucial. Changing national NHS culture is a major task but without promoting standardised practice, local factors on individual wards will continue to impact deprescribing efforts. This inconsistency can lead to unnecessary polypharmacy, distress for patients, and increased workload for the MDT. Although good practice exists, further dissemination, research and funding are needed to support all AMH patients in reducing inappropriate benzodiazepine/z-hypnotic use. Addressing underlying NHS pressures is essential to break the cycle of harmful polypharmacy and escalating patient behaviours.

## Supplementary Information

Below is the link to the electronic supplementary material.

Supplementary Material 1

## Data Availability

The qualitative data generated from this research project is not suitable for sharing as per ethical approval and the study protocol. If further information is required around the dataset, please contact [sonia.filmer1@nhs.net](mailto: sonia.filmer1@nhs.net) .

## Abbreviations

Benzos: Benzodiazepines
UK: United Kingdom
GABA: Gamma-aminobutyric acid
Z-hypnotics: Zopiclone/zolpidem
AMH: Adult mental health
NICE: National Institute for Heath and Care Excellence
NHS: National Health Service
PRN: As-needed
RCT: Randomised controlled trials
COREQ: Consolidated Criteria for Reporting Qualitative studies
CQC: Care Quality Commission
CMHT: Community mental health team
EPMA: Electronic prescribing systems
EUPD: Emotionally unstable personality disorder
MDT: Multi-disciplinary team
NMP: Non-medical prescriber
GPs: General Pratitioners
