# Supplementary material for: Facilitators and barriers to the deprescribing of benzodiazepines and Z-drug hypnotics in patients under 65 on adult mental health wards
Source: Sci Rep. 2025 Nov 21;15:41420. doi: 10.1038/s41598-025-25261-4 (PMC12639134; doi:10.1038/s41598-025-25261-4)
Supplement: Supplementary file 1 — Supplementary Material 1 [file 41598_2025_25261_MOESM1_ESM.docx]

**COREQ Checklist** ^1^

**Domain 1: Research Team and Reflexivity**

**1. Which author/s conducted the interview or the focus group?** The interviews were conducted by Sonia Filmer (SF). Ian Maidment (IM) provided supervision.

**2. What were the researchers’ credentials?** SF and IM are experienced mental health pharmacists. SF is undertaking this project as a research MSc with Aston University. IM is an experienced academic and NHS researcher and holds a PhD. IM has led four qualitative projects funded by NIHR and supervised many student projects.

**3. What was their occupation at the time of the study?** SF is a senior mental health pharmacist employed at Tees, Esk and Wear Valley NHS Foundation Trust. IM is a professor in Clinical Pharmacy, Aston University.

**4. Was the researcher male or female?** SF is female, IM is male.

**5. What experience or training did the researcher have?** SF is experienced in care of patients admitted to adult mental health wards and prior to this had an extensive career in primary care, including developing benzodiazepine withdrawal clinics. IM is experienced in mentoring those undertaking research. IM has led four qualitative projects funded by NIHR and supervised over ten master’s student’s projects.

**6. Was a relationship established prior to study commencement?** SF worked with some of the interviewees in her professional capacity prior to the commencement of the study. SF formally introduced herself as the researcher, clarifying this role and the topic before obtaining consent and commencing the interview.

**7. What did the participants know about the researcher?** The participants knew that SF was a pharmacist at Tees, Esk and Wear Valley NHS Foundation Trust undertaking this study as a researcher with Aston University as part of her master’s study. They also knew that SF had support from Aston University (from IM).

**8. What characteristics were reported about the interviewer/facilitator?** SF reported that she was an experienced pharmacist and researcher for the study.

**Domain 2: Study Design**

**9. What methodological orientation was stated to underpin the study?** An exploratory, qualitative study was conducted employing semi-structured interviews. Data were analysed using an inductive approach, acknowledging potential for SF to have clinical preconceptions, using thematic analysis within a grounded theory framework.

**10. How were participants selected?**  From advertising to existing professional contacts of SF and IM, advertising on social media and via the College of Mental Health Pharmacist. Also, through snowballing effect.

**11. How were participants approached?** Potential participants were asked to email SF to express and interest to take part. A Participant Information Sheet explaining the study was sent to the potential participants. Participants were not reimbursed financially but were offered a participation certificate. No participants requested a participation certificate

**12. How many participants were in the study?** The final sample comprised of twenty-nine participants

**13. How many people refused to participate or dropped out?** none

**14. Where was the data collected?** The interviews were carried out online on Microsoft teams. 3 interviews were completed in a private office at the researcher’s place of work, with the rest carried out from the researcher’s home. Most interviewees were in their workplace for the interviews, with the exception of two who participated in their online interview from home.

**15. Was anyone else present besides the participants and researchers?** No

**16. What are the important characteristics of the sample?** Health care professionals with experience of working on adult mental health inpatient wards within the last 6 months and were involved in reviewing benzodiazepines/hypnotics. Ethnicity data was collected.

**17. Were questions, prompts, guides provided by the authors?** **Was it pilot tested?** The interview schedule consisted of open-ended questions to avoid leading participants’ responses. Prompts were built into the schedule and employed if required. SF developed the interview schedule with input from IM. The interview was piloted with two healthcare professionals and the results from the pilot were included in the final dataset.

**18. Were repeat interviews carried out?** No

**19. Did the research use audio or visual recording to collect the data?** Interviews were audio and visually recorded on Microsoft Teams, which was also used to transcribe the date. This was then transcribed verbatim by SF into Word documents.

**20. Were field notes made during and/or after the interview or focus group?** Field notes were made during the interviews.

**21. What was the duration of the interviews or focus group?** The interviews were twenty to fifty minutes in length.

**22. Was data saturation discussed?** Data saturation was reached and discussed by SF with IM.

**23. Were transcripts returned to participants for comment and/or correction?** Transcripts were not returned to the participants, so member checking was not carried out. Participants were asked if they wished to receive a summary of the research report when the study was concluded.

**Domain 3: Analysis and Findings**

**24. How many data coders coded the data?** SF reviewed and coded the transcripts. SF discussed her findings with IM. SF analysed the data to identify the similarities and differences between the interview transcripts and to develop a set of themes which represent the whole data set. This was done on NVIVO software. SF discussed her approach to the analysis and themes identified with IM.

**25. Did authors provide a description of the coding tree?** No, but codes were represented in a wheel diagram.

**26. Were themes identified in advance or derived from the data?** Themes were derived from the data. Four key themes were elicited from the data with subthemes within these three themes.

**27. What software, if applicable, was used to manage the data?** Interviews were recorded on and transcribed by Microsoft Teams, then this transcript was copied into a Word document so it could be reviewed by SF. NVivo software was used for coding and analysing the data. The coded data was manipulated within Word documents.

**28. Did participants provide feedback on the findings?** Participants did not provide feedback.

**29. Were participant quotations presented to illustrate the themes/ findings? Was each quotation identified?** Participant quotes from the interview transcripts are presented in the results section. Each quotation is identified, anonymity was maintained, and each participant was given a pseudonym.

**30. Was there consistency between the data presented and the findings?** Yes, there was consistency between the data presented and the findings.

**31. Were major themes clearly presented in the findings?** The four themes are presented with representative quotations which are discussed in the results and discussion section.

**32. Is there a description of diverse cases or discussion of minor themes?** Yes, diverse cases have been discussed and minor themes are also discussed where pertinent to the research.

#

# **Advertising flyer**

**FABDOB Study**

**Facilitators And Barriers to the De-prescribing Of Benzodiazepines and Z-drug Hypnotics in patients under 65 on Adult Mental Health Wards**

Would you like to take part in a research project looking at factors which may prevent or encourage the review of benzodiazepine and hypnotic medication on adult mental health wards?

Aston university is conducting a study funded by the NIHR. We would like to interview health care professionals (including medical staff, mental health nurses and pharmacists) who have worked on NHS adult mental health wards with patients under 65 within the last 6 months.

Interviews will be conducted via video conferencing e.g., Microsoft Teams and may last up to 60 minutes.

**Who is doing the research? More information?**

The research is being carried out by Sonia Filmer. The supervisor is Professor Ian Maidment. If you would like more information or to take part, please contact Sonia (180204706@aston.ac.uk) or Ian ([i.maidment@aston.ac.uk](mailto:i.maidment@aston.ac.uk)) directly via email.

#

# **Participant Information Sheet (NHS recruitment)**

**Facilitators And Barriers to the De-prescribing Of Benzodiazepines and Z-drug Hypnotics in patients under 65 on Adult Mental Health Wards (FABDOB)**

**Invitation**

We would like to invite you to take part in a research study forming part of a master’s project for Sonia Filmer, funded by the National Institute for Health Research.

Before you decide if you would like to participate, take time to read the following information carefully and, if you wish, discuss it with others such as your family, friends or colleagues.

Please ask a member of the research team, whose contact details can be found at the end of this information sheet, if there is anything that is not clear or if you would like more information before you make your decision.

**What is the purpose of the study?**

The study will try to identify barriers to the reviewing of benzodiazepines and z drug hypnotics on adult mental health wards. It will also try to identify what helps in the review and reduction of prescribing of these medicines. This may then encourage further research to see what could be done to support staff in reviewing of benzodiazepines and z-drug hypnotics. The study involves conducting interviews with health care professionals who are involved in the review of benzodiazepines and z-drug hypnotics on adult mental health wards.

**Why have I been invited?**

You are being invited to take part in this study because you are a health care professional (for example mental health nurse, doctor or pharmacist) who has worked on an NHS adult mental health ward, with patients aged between 18 and 65, within the last 6 months and has experience in the use and review of benzodiazepines and z-drug hypnotics.

**What will happen to me if I take part?**

If you agree to take part, you will be interviewed online. You will be asked to sign a consent form before taking part. By consenting, you are agreeing to the use of your interview data for appropriate analysis within the overall scope of the project. Participation would involve a single interview, conducted by Sonia Filmer. This would last up to 60 minutes and take place at a time that suits you. Interviews will cover a range of topics related to the review of benzodiazepines and hypnotics in a ward environment. The interview would, with your consent, be audio and visually recorded. Sonia Filmer will also ask you some background information about yourself, such as your role and experience.

**Do I have to take part?**

**No.** It is up to you to decide whether or not you wish to take part.

If you do decide to participate, you will be asked to provide informed consent via a form, which will be emailed to you prior to the interview for you to sign electronically and return via email.

You can halt your participation in the research interview at any time by telling the researcher, Sonia Filmer and any data collected up to that point will not be used. If you wish to withdraw your data after participation, then you have up to 14 days to do so by contacting Sonia Filmer via email and giving your participant number. After this point, your data will be anonymised and it will not be possible to withdraw it.

**Will my taking part in this study be kept confidential?**

**Yes.** A code will be attached to all the data you provide to maintain anonymity. Analysis of your data will be undertaken using coded data.

The data we collect will be stored electronically on a secure, encrypted password protected computer server or secure cloud storage device.

To ensure the quality of the research Aston University may need to access your data to check that the data has been recorded accurately e.g., for the purposes of audit.

**How will the conversation during the interview be recorded and the information I provide managed?**

With your permission we will audio record the interview and take notes. Recordings will either be on Microsoft Teams, an NHS approved Dictaphone or an encrypted Dictaphone, depending on your preference and the Information Governance requirements of the NHS trust you work for.

The recording will be typed into a document (transcribed) by a member of the research team/transcriber approved by Aston University. This process will involve removing any information which could be used to identify individuals e.g., names, locations etc.

Audio recordings will be destroyed as soon as the transcripts have been checked for accuracy.

We will ensure that anything you have told us that is included in the reporting of the study will be anonymous.

You, of course, are free not to answer any questions that are asked without giving a reason.

**What happens if I tell you something that concerns you about my health or welfare or that of the people under my care?**

In the unlikely event of this happening, we will discuss with you how this should be addressed. If necessary, to protect you and the people you care for, we will report your concern to the appropriate person or bodies.

**What are the possible benefits of taking part?**

The study may not directly benefit you. However, we often find people benefit from sharing their thoughts in projects like this. The research goal is to identify barriers and facilitators to the deprescribing of benzodiazepine and z-drug hypnotics with the aim of encouraging further research in this area to see if support can be given to healthcare practitioners around this. If you provide your email address on the consent form, you will receive a lay summary of the research report. This will include recommendations about ways that you, as practitioners, might benefit in the future.

If requested, you can be provided with a copy of a non-accredited certificate of participation, which you can use as evidence for your continued professional development that you have taken part in some reflective practice around the review benzodiazepine and z-drug hypnotics.

**What are the possible risks and burdens of taking part?**

We believe the risks from taking part are low. There may be issues raised in the discussion I have with you that are sensitive or distressing to you. In this unlikely situation, you will not have to answer any questions that you don’t want to. You may also take a break from the discussion at any time and return to it at a later convenient time to you if you would prefer. You can stop the interview at any time.

**What will happen to the results of the study?**

The results of this study will form part of a master’s project and may be published in scientific journals and/or presented at conferences. If the results of the study are published, your identity will remain anonymous. A lay summary of the results of the study can be forwarded to you when the study has been completed. Should you wish to receive a copy, please provide your email address on the consent form, or contact a member of the research team. The anonymised results may be shared with the funder.

**Expenses and payments**

There are no expenses available for taking part in this study.

**Who is funding the research?**

The master’s study is being funded by the National Institute for Health Research (NIHR).

**Who is organising this study and how is my data being used?**

Aston University is organising this study and acting as data controller for the study. Research data will be used only for the purposes of the study or related uses identified in this information sheet or appendix A.

**Who has reviewed the study?**

The study has been given a favourable ethical opinion by Aston University Research Ethics and Integrity Committee and approved by the Health Research Authority.

**What if I have a concern about my participation in the study?**

If you have any concerns about your participation in this study, please speak to the research team and they will do their best to answer your questions. Contact details can be found at the end of this information sheet.

If the research team are unable to address your concerns or you wish to make a complaint about how the study is being conducted you should contact the Aston University Research Integrity Office at [research_governance@aston.ac.uk](mailto:research_governance@aston.ac.uk) or via the University switchboard on +44 (0)121 204 3000.

**Research team**

Student: Sonia Filmer. Email at 180204706@aston.ac.uk

Project Supervisor: Professor Ian Maidment, College of Health and Life Sciences, Aston Pharmacy School, Aston University. Email: [i.maidment@aston.ac.uk](mailto:i.maidment@aston.ac.uk)

Appendix A

**Transparency statement**

**How will we use information about you?**

We will need to use information from you for this research project.

This information will include your name, job title, the NHS trust you work for and email address. People will use this information to do the research or to check your records to make sure that the research is being done properly.

People who do not need to know who you are will not be able to see your name or contact details. Your data will have a code number instead.

We will keep all information about you safe and secure.

Once we have finished the study, we will keep some of the data so we can check the results. We will write our reports in a way that no-one can work out that you took part in the study.

**What are your choices about how your information is used?**

- You can stop being part of the study at any time, without giving a reason.
- You can withdraw previously collected data up to 14 days after the interview, otherwise data will be retained.
- We need to manage your records in specific ways for the research to be reliable. This means that we won’t be able to let you see or change the data we hold about you.

**Where can you find out more about how your information is used?**

You can find out more about how we use your information

- at [www.hra.nhs.uk/information-about-patients/](https://www.hra.nhs.uk/information-about-patients/)
- our webpage available at **www.aston.ac.uk/dataprotection**
- by asking one of the research team or
- by sending an email to [**dp_officer@aston.ac.uk**](mailto:dp_officer@aston.ac.uk)

#

# **Consent Form**

**Facilitators And Barriers to the De-prescribing Of Benzodiazepines and Z-drug Hypnotics in patients under 65 on Adult Mental Health Wards (FABDOB)**

**Name of Chief Investigator: Ian Maidment**

**Please initial boxes**

|  | I confirm that I have read and understand the Participant Information Sheet (REC ID: [HLS21095] IRAS number 322529, Version 2, 16/5/23) for the above study. I have had the opportunity to consider the information, ask questions and have had these answered satisfactorily. |  |
| --- | --- | --- |
|  | I understand that I am able to withdraw my data up to 14 days after taking part in the study by contacting Sonia Filmer, after this time my data will be anonymised and I will no longer be able to withdraw. |  |
|  | I agree to my personal data and data relating to me collected during the study being processed as described in the Participant Information Sheet. |  |
|  | I understand that if during the study I tell the research team something that causes them to have concerns in relation to my health and / or welfare, they may need to breach my confidentiality. |  |
|  | I agree to my interview being audio recorded and to anonymised direct quotes from me being used in publications resulting from the study. |  |
|  | I agree to take part in this study. |  |

_________________________ ________________ ___________________

Name of participant Date Signature

___________________________ ________________ ___________________

Name of Person receiving consent. Date Signature

| If you wish to receive a lay summary of the research project upon its completion, please provide an email address to which the summary can be sent. |
| --- |
| **Email Address:** |

#

# **Demographic Data Form**

Facilitators And Barriers to the De-prescribing Of Benzodiazepines and Z-drug Hypnotics in patients under 65 on Adult Mental Health Wards (FABDOB)

Some Questions About You

In order for me to learn about the range of people taking part in this research, I would be grateful if you could answer the following questions. Please either write your answer in the space provided or circle or tick the answer (or answers) that best applies to you.

| 1 | What is your current job title? |  | | | |
| --- | --- | --- | --- | --- | --- |
| 2 | Gender.  Do you identify as: | o A woman  o A man  o Non-binary  o Self-defining (please state) ___________________  o Prefer not to say | | | |
| 3 | I am: | Full-time employed | Part-time employed | | Other:_______________ |
| 4 | How long have you been in your current role? | | | ___________________________ years | |
| 5 | How long have you worked in the mental health sector for? | | |  | |
| 6 | Which region of the U.K. do you currently work in or have you worked in within the last 6 months. | | |  | |
| 7 | What is your ethnic group? | | | White  Mixed or multiple ethnic groups  Asian or Asian British  Black, African, Caribbean or Black British  Other ethnic group  or  Prefer not to say | |

# **Interview Guide**

Could you describe for me your current role?

What job do they do?

What type of ward do they work on?

What is your experience of the prescribing, use and review of benzodiazepines (such as lorazepam/ diazepam) and hypnotics (such as zopiclone)?

Both previous experience

Recent more current experience

Can you tell me about how benzodiazepines and hypnotics are reviewed and monitored in your current place of work? (on the last acute adult ward you worked on)

Whose role is it to ensure they are reviewed?

Who monitors whether someone is over-sedated including the next morning hangover?

What do you understand de-prescribing to be? (if unsure: de-prescribing is the process of withdrawal of an inappropriate medication, supervised by a health care professional, with the goal of managing polypharmacy and improving outcomes)

How common, or otherwise, is the deprescribing of benzodiazepines and hypnotics?

Is de-prescribing regularly discussed in MDT meetings e.g., report outs, consultant ward rounds? Is there a formal process, for example, or any guidelines?

What do you think gets in the way of, or is a barrier to, the de-prescribing of benzodiazepines and hypnotics?

What helps in the de-prescribing of benzodiazepines and hypnotics in your clinical environment?

Whose responsibility is it to ensure that, if appropriate, de-prescribing occurs? Do you have any suggestions for what could be done to improve the review process of benzodiazepine and hypnotic medication?

In the ideal world, what would you like to see happen?

Realistically, what do you think might help bearing in mind the pressures in your current clinical environment?

As you approach discharge, and throughout the pre and post discharge period, what, if any, actions are taken around the prescribing or deprescribing of benzodiazepines and hypnotics?

Thank you for taking part in this interview. Do you have anything else you would like to add around what we have discussed today?
